# Oral health-related quality of life in primary Sjögren's Syndrome

**DOI:** 10.4317/medoral.26934

**Published:** 2025-01-26

**Authors:** Lia Ingolotti, Marisel Bejarano, Natalia Tamborenea, Aixa Mercé, Constanza Arguissain, Luz Martin, Julieta Morbiducci, Victoria Azcona, Leandro Teplizky, Eugenia Casals, Adriana Hernandez, Mariana Marseillan, Anastasia Secco

**Affiliations:** 1 Chief Resident at B. Rivadavia General Acute Hospital, Rheumatology Section, Buenos Aires, Argentina; 2 Staff Physician at B. Rivadavia General Acute Hospital, Rheumatology Section, Buenos Aires, Argentina; 3 Staff Physician at Dr. Ramón Carrillo Dental Hospital, Dentistry, Buenos Aires, Argentina; 4 Staff Physician at B. Rivadavia General Acute Hospital, Dentistry, Buenos Aires, Argentina; 5 Chief of Section at B. Rivadavia General Acute Hospital, Rheumatology Section, Buenos Aires, Argentina

## Abstract

**Background:**

Primary Sjögren Syndrome (pSS) is an autoimmune disease that usually affects salivary glands. Research about the impact of oral health in quality of life of patients with pSS is scarce. Objectives: to describe the characteristics of oral involvement in patients with pSS; To assess quality of life related to oral health (QOL-OH); to determine association between QOL-OH and saliva production, disease activity, and damage.

**Material and Methods:**

An observational, analytical and cross-sectional study was conducted. Patients aged ≥18 years with pSS were included. Primary outcome was assessed by the Oral Health Impact Profile (OHIP14sp). The EULAR Sjogren's Syndrome Patient Reported Index (ESSPRI), the EULAR Sjögren's syndrome Disease Activity Index (ESSDAI), the Sjogren's Syndrome Damage Index (SSDI) and a Visual Analogue Scale (VAS) for xerostomia, were performed. A dentist evaluated the Decayed, Missing, and Filled Permanent Teeth index (DMFT), O'Leary index (OLI) and Loe & Silnes index (LSI). A multiple linear regression model was performed, taking OHIP14sp as the dependent variable.

**Results:**

51 patients were included. Mean age 54 (±13 years). The OHIP-14sp median was 16 [6-25], xerostomia VAS median was 60 [30-80]. Mean of ESSPRI: 4 (± 2.6), ESSDAI median: 0 [0-2], SSDDI median: 3 [2-4]. Oral involvement occurred in 100% of patients, DMFT median: 22 [14-28], OLI median: 21[13-30]. In the univariate analysis, OHIP14sp was significantly associated with ESSPRI (β2 95%CI 0.72-3.3), xerostomia VAS (β0.19 95%CI 0.08-0.29) and category 2 of the LSI (β: 18 95% CI: 5-31). In the multivariate analysis, OHIP14sp was independently and significantly associated with xerostomia VAS (β0.19 95%CI 0.09-0.29) and category 2 of LSI (β19 95% CI: 7.7-29.7).

**Conclusions:**

These findings demonstrate the effects of xerostomia on daily life of patients influencing not only their oral health but also their quality of life.

** Key words:**Sjögren´s syndrome, quality of life, oral health impact profile.

## Introduction

Primary Sjögren's syndrome (SS) is an autoimmune rheumatic disease that mainly affects the salivary and lacrimal glands due to different degrees of lymphocytic infiltration, but systemic manifestations can also be found. The prevalence in the general population has been estimated at 0.01% - 3.00%, depending on the population studied and classification criteria used (1,2).

There are a variety of oral manifestations resulting from salivary gland dysfunction, such as an increased risk of dental caries refractory to treatment, loss of teeth and a greater number of cariogenic microorganisms (3). Many studies have shown a direct relationship between severity of xerostomia and deterioration in oral health. In contrast, there is no a clear increase in the incidence or severity of periodontal disease in patients with SS (4).

Over time, different tools were developed to assess the activity, damage and quality of life related to this disease: ESSPRI (EULAR Sjogren's Syndrome Patient Reported Index), ESSDAI (EULAR Sjögren's Syndrome Disease Activity Index) and SSDDI (Sjögren's Syndrome Disease Damage Index), among others. The Oral Health Impact Profile (OHIP) was validated as a generic self-administered questionnaire to assess quality of life related to oral health. The original version of the OHIP was developed in Australia, in 1994, by Slade & Spencer and is made up of 49 items (OHIP-49) (5). The OHIP-14sp is an abbreviated version in Spanish, which consists of 14 questions and evaluates seven dimensions including functional limitation, physical pain, physical disability, psychological disability, social disability and handicap in the last 12 months. It was validated in Spanish by Montero-Martín J *et al*. in 2009 reflecting a high level of reliability (6). It was also validated and adapted in Chile, in a population without pSS (7).

Our data reflects that there is a significant prevalence of this disease and the availability of scientific works that provide information about how oral health affects the quality of life of patients is limited. This study was carried out with the purpose of generating behaviors that contribute to improving the health of patients with pSS.

- Objectives: 1- To describe, through an objective evaluation by a dentist, the characteristics of oral involvement in patients with pSS. 2- To assess quality of life related to oral health. 3- To determine association between quality of life related to oral health and saliva production, disease activity and damage in patients with pSS.

## Material and Methods

An observational, analytical and cross-sectional study was conducted.

Patients were recruited from the Rheumatology section of Bernardino Rivadavia Hospital of the Autonomous City of Buenos Aires (C.A.B.A.), Argentina, who were referred for dental care to the dentistry section in the same hospital. Patients were included if they met the classification criteria of pSS according to American - European 2002 and/or ACR-EULAR 2016, they were 18 years or older and were able to comply with the visits and procedures of the protocol and sign the informed consent.

Patients were excluded if they had another associated autoimmune rheumatic disease as Rheumatoid Arthritis, Systemic Lupus Erythematosus, Dermatomyositis, Polymyositis or Vasculitis; other conditions that may favor the presence of cavities: immunosuppression due to causes other than Sjögren's Syndrome, physical disability that may make dental hygiene difficult and/or use of orthodontic devices (fixed or removable); previous head and neck irradiation, active hepatitis C virus infection (confirmed by PCR), acquired immunodeficiency syndrome (AIDS), sarcoidosis, amyloidosis, graft versus host reaction, systemic disease associated with IgG4. Patients with difficulty reading and understanding the Spanish language were also excluded.

A dentist evaluated oral health based on the Decayed, Missing, and Filled Permanent Teeth index (DMFT) which quantifies the prevalence of dental caries and its result is obtained by the sum of decayed, missing and filled permanent teeth, and is interpreted as follows: 0 - 1.1: very low prevalence; 1.2 - 2.6: low; 2.7 -4.4: moderate; 4.5 - 6.5: high; and 6.6 and more: very high.) (8), the Loe and Silnes Index (measures the thickness and extension of the plaque deposited on the tooth surface, categorized into 0,1,2 and 3, code 3 represents the greatest thickness and extension) (9) and oral quality of life in the last 12 months: measured by the OHIP-14sp (the responses were quantified on the Likert scale with values from 0 to 4, where 0 represents the lowest value and 4 is the highest, the total score from 0 to 56 points where the highest scores represent greater negative impact of the condition) (6,7).

A rheumatologist evaluated the clinical characteristics of disease activity by the ESSPRI (10), ESSDAI (11) CLINESSDAI (12) and damage using SSDDI (13). Additionally, the Xerostomia Visual Analogue Scale (VAS) was performed (with a score of 0-100 mm where the closest to 100 mm means the worst discomfort). Unstimulated salivary flow measurement (sialometry) was also performed, if the flow rate was ≤ 1,5 ml/15 minutes it was considered positive. The total flow rate was assessed too. Other laboratory parameters evaluated were AntiNuclear antibodies (ANA), anti-RO and anti-La autoantibody using ELISA assays. They were reported as positive or negative and titers were also analyzed.

Until the date of preparation of this research work, no studies were found that evaluate oral quality of life as a primary objective, which is why it was decided to carry out a pilot test with a minimum of 30 patients.

This research was approved by the Research Ethics Committee (CEI) of the Bernardino Rivadavia Hospital, and respected and complied with all relevant legislation and regulations to which the CEI adhered according to its Operational Procedures Manual (POE). The confidentiality of the personal information recorded in the medical history was respected.

- Statistical analysis: Continuous variables were described as mean and standard deviation (SD) or median and interquartile range (IQR), depending on their distribution. Categorical variables were expressed in percentages. Univariate and multivariate linear regression analysis was performed taking the quality of life related to oral health as the dependent variable adjusted for possible confounders. The performance of the model was evaluated.

## Results

51 patients were included, 98% were female, mean age 54±13 years. The median OHIP-14sp was 16 (IQR 6-25). Although the dentist examined the patients for oral lesions, no ulcers, oropharyngeal candidiasis or other types of lesions were found, and only active odontogenic infectious processes were observed. The median VAS for xerostomia was 60 (IQR 30-80). The mean of the ESSPRI was 4± 2.6, the median of the ESSDAI was 0 (IQR 0-2), the median of the SSDDI was 3 (IQR 2-4). Sialometry was positive in 75% and, of them, the median in milliliters was 0.7 (IQR 0.5-1). The ANAs were positive in 90% and the anti-Ro antibodies were positive in 74.5%. Regarding treatment, 56% were treated with conventional immunosuppressants (of which 75% were hydroxychloroquine), 4% with biologicals and 9.8% with glucocorticoids with a median dose of 10 mg/day (IQR 5-20). 60% used treatment for xerostomia (M agonists or salivary substitutes).

According to the dental evaluation, oral involvement occurred in 100%, the median DMFT was 22 (IQR 14-28). Poor dental condition was detected in 88.2% with a median O'Leary index of 21 (IQR 13-30). The Loe & Silnes index was 1 in 51%, 2 in 18.9% and 3 in 8.1%. The population characteristics are summarized in Table 1.

In the univariate analysis, OHIP14sp was significantly associated with the ESSPRI (β coefficient: 2.04. 95% CI 0.72-3.35), the xerostomia VAS (β coefficient 0.19, 95% CI 0.08-0. 29) and category 2 of the Loe & Silnes index (β coefficient: 18. 95% CI: 5-31). No significant association was found with ESSDAI, SSDDI or sialometry (Table 2).

In the multivariate analysis, OHIP14sp was independently and significantly associated with the xerostomia VAS (β coefficient: 0.19 95%CI 0.09-0.29) and category 2 of the Loe & Silnes index (β coefficient: 19 95% CI 7.7-29.7).

## Discussion

This study evaluated oral health and its impact on quality of life in 51 patients with pSS. Significant association was found between oral health problems, xerostomia severity and quality of life, with a major effect on xerostomia.

Fernández Castro *et al*., conducted a cross-sectional study in patients with this disease; OHIP14sp, an oral health questionnaire, DMFT index and a visual analogue scale of xerostomia were performed. It was reported that more than 50% of patients evaluated had oral injuries. Oral health impacted negatively in the quality of life of patients, being worst in those patients with objective pathological oral signs, and in those with peripheral nervous system involvement (14). These findings are in line with those we obtained in our study. It should be taken into account that we analyzed patients as well with ACR-EULAR 2016 classification criteria of pSS and also evaluated the impact of auto-antibody levels. Fernández Castro *et al*. found that seronegative patients had higher scores in the OHIP, but this has not been confirmed in our study.

Published studies had observed that oral ulcers, difficulty in speaking and dysphagia are the manifestations with the most negative impact in the quality of life of these individuals (15,16). No ulcers, oropharyngeal candidiasis or other types of lesions (except those infections of odontogenic origin) were reported in our investigation; therefore, an analysis of this aspect was not performed.

The impact of oral health in the global quality of life of patients with pSS have been also reported. The publication by Enger TB *et al*., a multicenter cohort of 246 patients, reported that higher scores in OHIP14 were strongly related to worse results in Short Form-36 Health Survey (SF 36) (15). The investigation carried out by Stewart *et al*. that included 39 patients with pSS, concluded that the impact on oral health was an independent risk factor for poor quality of life, evaluated by SF 36, suggesting that a proactive management of this aspect, could improve significantly the quality of life of these patients (17). In our study, oral health-related quality of life was assessed using a specific questionnaire; the overall quality of life was not evaluated taking into account other aspects included for example in SF 36. We are aware that addressing the quality of life and functionality of patients with a high burden of disease during daily consultation is crucial.

Additionally, there are some reports in the literature describing molecular changes in saliva samples and oral signs in patients with pSS. Azuma, Naoto *et al*. have demonstrated a proportional relationship between the amount of cytoprotective molecular components in saliva, such as Epidermal Growth Factor (EGF), and the presence of oral manifestations as measured by the Oral Health Impact Profile (OHIP) (18). It has been hypothesized that EGF contributes to the resolution of inflammatory intraoral lesions. Although we did not analyze the cytochemical aspects in our study, we consider that measuring these parameters could be important for a more comprehensive understanding of their role in oral manifestations and the overall management of pSS.

One of the main advantages of our study is that it takes into account variables which are often overlooked in similar researches. Factors such as the use of salivary substitutes, muscarinic agonists, immunosuppressors, sialometry, presence of positive anti-Ro autoantibodies and anti-Ro autoantibody titer were considered. The inclusion of these variables provides a more complete understanding of the clinical context and improves our view on oral manifestations in patients with pSS, offering a more detailed and accurate perspective than that commonly found in the literature. This study had some limitations, including the absence of a second observer for dental findings, the lack of unstimulated salivary flow measurements, and the absence of a control group without pSS, it is noteworthy that no data were available in our setting at the time of the study.

## Conclusions

Our study highlights the critical importance of early dental assessment and intervention for patients with pSS, emphasizing the need for interdisciplinary collaboration among healthcare professionals to prevent oral damage and improve patient outcomes. The findings revealed that all patients in the study had oral involvement, with a deteriorated oral quality of life, closely linked to the degree of xerostomia. This underscores the significance of integrating dental care to enhance oral hygiene and condition. Although our analysis did not measure general quality of life or establish precise associations regarding the onset of oral involvement or the impact of interventions, we believe that early detection and treatment of oral manifestations could greatly enhance the long-term quality of life for patients with pSS.

## Figures and Tables

**Table 1:** Clinical and demographic characteristics.

Variables	Results
Female sex (%)	98,04
Mean age in years	54,7 ± 13,6
Time of evolution of the disease measured in months. Median (IQR)	60 (13-132)
Alcoholism (%)	5,8
Current smoking (%)	3,9
Use of drugs that can cause SICCA (%)	33,4
ANAs + >1/80 (%)	90,2
ANA autoantibody titer: Median (IQR)	1/640 (1/320-1/1280)
Anti Ro autoantibody titer (ELISA AU). Median (IQR)	102,5 (82-123)
Anti-La autoantibody titer (ELISA AU) Median (IQR)	68 (40-88)
Use of DMARDc (%)	56
Muscarinic agonist drugs use (%)	92,16
Salivary substitute use (%)	29,4
Glucocorticoid dosage. Median (IQR)	10 (5-20)
OHIP 14sp. Median (IQR)	16 (6-25)
xerostomia VAS. Median (IQR)	60 (30-80)
ESSPRI. Mean ± SD	4±2,6
ESSDAI. Median (IQR)	0 (0-2)
SSDDI. Median (IQR)	3 (2-4)
Unstimulated sialometry resulting positive (%)	75
Unstimulated sialometry in milliliters. Median (IQR)	0,7 (0,5-1)
DMFT. Median (IQR)	22 (14-28)
O´Leary. Median (IQR)	21 (13-30)
Loe & Silness: Cat 1 (%)	51
Loe & Silness: Cat 2 (%)	18,9
Loe & Silness: Cat 3 (%)	8,1
Dental status: Good (%)	3,92
Dental status: Regular (%)	7,84
Dental status: Poor (%)	88,24

References: IQR (Inter Quartile Range) SD (Standard Deviation) ANAs (AntiNuclear Antibodies) ELISA AU (Enzyme-Linked ImmunoSorbent Assay Arbitrary Units) DMARDc (Conventional Disease-modifying antirheumatic drugs) OHIP 14sp (Oral Health Impact Profile) VAS (visual analog scale) ESSPRI (EULAR Sjögren's Syndrome Patient Reported Index) ESSDAI (EULAR Sjögren's Syndrome Disease Activity Index) SSDDI (Sjögren Syndrome Disease Damage Index) DMFT (number of Decayed, Missing, and Filled Permanent Teeth).

**Table 2:** Results of univariate analysis between OHIP14sp and independent variables.

Analyzed variable	β Coefficient	95% CI	*p value*
Sex	12,4	-13,9-38,8	0,347
Age	0,05	-0,22-0,32	0,710
Time of evolution of the disease	-0,01	-0,05-0,039	0,791
Alcoholism	0,10	-15,6-15,81	0,989
Smoking	-1,81	-20,8-17,2	0,850
Salivary substitute use	4,2	-3,8-12,2	0,298
Muscarinic agonist use	4,8	-8,8-18,6	0,475
DMARDc use	3,13	-4,4-10,66	0,408
Positive Sialometry	4,8	-3,12-12,85	0,227
ANA positive	2,48	-9,92-14,88	0.690
Positive Anti Ro autoantibody	1,55	-6,91-10,02	0,714
Anti-Ro autoantibody titer	-0,01	-0,03-0,009	0,253
Xerostomia VAS	0,18	0.08-0,29	0,000
ESSPRI	2,03	0,72-3,35	0,003
ESSDAI	0,40	-0,60-1,39	0,43
SSDDI	0,68	-3,14-4,5	0,716
DMFT	0,14	-0,31-0,59	0,534
O´Leary	0,17	-0,07-0,41	0,157
Loe & Silness (1)	3,9	-4,9-12,9	0,374
Loe & Silness (2)	18,7	7,7-29,8	0,002
Loe & Silness (3)	13,7	0,65-28	0,061
Good dental status	2,4	-5,6 - 10,4	0.549
Regular dental status	20	-2,3-42,3	0,078
Poor dental status	13,2	-5,4-31,9	0,161

References: CI (Confidence Interval). ANAs (AntiNuclear Antibodies). DMARDc (Conventional Disease-modifying antirheumatic drugs). OHIP 14sp (Oral Health Impact Profile) VAS (visual analog scale) ESSPRI (EULAR Sjögren's Syndrome Patient Reported Index) ESSDAI (EULAR Sjögren's Syndrome Disease Activity Index) SSDDI (Sjögren Syndrome Disease Damage Index) DMFT (number of Decayed, Missing, and Filled Permanent Teeth).
